# Structure of the Vesicular Stomatitis Virus N^0^-P Complex

**DOI:** 10.1371/journal.ppat.1002248

**Published:** 2011-09-22

**Authors:** Cédric Leyrat, Filip Yabukarski, Nicolas Tarbouriech, Euripedes A. Ribeiro, Malene Ringkjøbing Jensen, Martin Blackledge, Rob W. H. Ruigrok, Marc Jamin

**Affiliations:** 1 UMI 3265 UJF-EMBL-CNRS Unit of Virus Host Cell Interactions, Grenoble, France; 2 UMR 5075 CEA-CNRS-UJF, Institut de Biologie Structurale, Grenoble, France; Institut Pasteur, France

## Abstract

Replication of non-segmented negative-strand RNA viruses requires the continuous supply of the nucleoprotein (N) in the form of a complex with the phosphoprotein (P). Here, we present the structural characterization of a soluble, heterodimeric complex between a variant of vesicular stomatitis virus N lacking its 21 N-terminal residues (N_Δ21_) and a peptide of 60 amino acids (P_60_) encompassing the molecular recognition element (MoRE) of P that binds RNA-free N (N^0^). The complex crystallized in a decameric circular form, which was solved at 3.0 Å resolution, reveals how the MoRE folds upon binding to N and competes with RNA binding and N polymerization. Small-angle X-ray scattering experiment and NMR spectroscopy on the soluble complex confirms the binding of the MoRE and indicates that its flanking regions remain flexible in the complex. The structure of this complex also suggests a mechanism for the initiation of viral RNA synthesis.

## Author Summary

The negative sense RNA genome of the rhabdoviruses is encapsidated by the nucleoprotein, and the replication of the genome requires a continuous supply of RNA-free, monomeric nucleoprotein (N^0^) to encapsidate the newly synthesized (+)RNA intermediate antigenomes and (−)RNA genomes. In this process, the viral phosphoprotein acts as a chaperone, forming a heterodimeric complex, named N^0^-P, which prevents nascent N molecules from self-assembling and from binding to cellular RNAs. We reconstructed the N^0^-P complex of the prototype rhabdovirus, vesicular stomatitis virus, and characterized its structure by crystal X-ray diffraction and solution experiments. Our results show how the N-terminal region of the phosphoprotein folds upon binding to the RNA-free nucleoprotein and how it prevents the non-specific encapsidation of host-cell RNA. This complex is soluble and heterodimeric, but by forcing it to polymerize into a crystal it associated into a circular decamer of heterodimers very similar to the previously crystallized decameric N-RNA ring. On the basis of our results, we propose a model that explains the role of the phosphoprotein in the encapsidation of newly synthesized RNA and in the initiation of RNA synthesis by the viral polymerase.

## Introduction

Negative-sense RNA viruses include numerous major human pathogens such as influenza virus, rabies virus, measles virus and respiratory syncytial virus. The (−)RNA genome of these viruses is condensed by a viral nucleoprotein (N) into a helical nucleocapsid [1], that associates with the polymerase complex and serves as the template for RNA replication and transcription [2]. Replication of the genome thus requires a continuous supply of N molecules to encapsidate both the (+)RNA intermediate copies and the newly synthesized (−)RNA genomes in single-stranded forms [3]. For non-segmented (−)RNA viruses of the *Rhabdoviridae* and *Paramyxoviridae*, N is assisted by the viral phosphoprotein (P). P binds to nascent RNA-free N, forming a N^0^-P complex (the superscript ^0^ denotes the absence of RNA) that prevents the polymerization of N and the non-specific encapsidation of host cell RNAs [4], [5], [6], [7], [8], [9]. These processes are independent of each other [10] and, therefore, P has to fulfill two chaperone activities; blocking both RNA binding and self-assembly of N. P is a modular protein comprising a long N-terminal disordered region and two folded domains, a central oligomerization domain and a C-terminal nucleocapsid-binding domain, separated by a flexible linker [11], [12]. The N^0^-binding region is localized in the N-terminal disordered region [7], [8], [9], and it has been demonstrated that in vesicular stomatitis virus (VSV), a prototypical rhabdovirus, this region of P contains transient helical elements and may thus constitute a short molecular recognition element (MoRE) that folds upon binding to its partner [13].

In rhabdovirus nucleocapsids, every N molecule binds nine nucleotides in a positively charged cavity at the interface between its N- (N_NTD_) and C-terminal (N_CTD_) domains [14], [15]. The N-RNA complex is stabilized by multiple salt bridges between the sugar-phosphate backbone of the RNA and basic residues of N, by contacts between neighboring N molecules involving hydrophobic side-to-side interactions, mainly between adjacent N_CTD_, and by the exchange of N- and C-terminal sub-domains between adjacent N protomers (N_NT_-arm, aa 1–21 and N_CT_-loop, aa 340–375, respectively) [14], [15]. Once formed, the N-RNA complex is stable and cannot be disassembled by full-length P [16]. However, on the basis of the N-RNA structure, we hypothesized that deletion of the N_NT_-arm may sufficiently destabilize the N-RNA complex so that P or a peptide fragment of P containing the MoRE that binds N^0^, could displace the RNA molecule.

In this study, we report the reconstitution of complexes between a recombinant N of VSV lacking the 21 N-terminal residues, N_Δ21_, and either full-length P dimer [11] or a peptide encompassing the N^0^-binding MoRE of P [13], named here P_60_, that comprises the first 60 amino acids of P, a two-amino acid linker and a C-terminal His_6_-tag. The characterization by absorbance spectroscopy and size-exclusion chromatography (SEC) combined with static light scattering (MALLS) demonstrates that both N_Δ21_^0^-P_60_ and N_Δ21_^0^-P dimer complexes are free of RNA in solution, forming soluble heterodimers or heterotrimers, respectively. Therefore, P_60_ fulfills both chaperone activities of full-length P. The heterodimer N_Δ21_^0^-P_60_ crystallized, but under the crystallization conditions, it assembled into a circular decamer of heterodimers very similar to the previously crystallized decameric N-RNA ring. The crystal structure of the decameric form of the N_Δ21_^0^-P_60_ complex reveals the molecular mechanisms by which the N^0^-binding MoRE of P attaches to N. NMR spectroscopy confirms that the MoRE of P binds to N in the N_Δ21_^0^-P_60_ complex in solution as in the crystal structure and shows that the regions of P flanking this MoRE remain flexible in the complex. Finally, these results suggest mechanisms for the encapsidation of newly synthesized RNA and for the initiation of RNA synthesis by the viral polymerase.

## Results

### Strategy for reconstituting the N_Δ21_^0^-P and N_Δ21_^0^-P_60_ complexes

Production of a mutant of N deleted of its 21 first N-terminal residues (N_Δ21_) in *Escherichia coli* led to the formation of inclusion bodies and of poorly soluble complexes, which could not be purified. In order to improve the solubility of the N_Δ21_ mutant, it was produced in *E. coli* in fusion with an N-terminal maltose binding protein (MBP) tag. The purified MBP-N_Δ21_ formed soluble, oligomeric N-RNA complexes, which eluted next to the exclusion volume of a Superdex S200 column (Figures S1A and S1B in Text S1). The presence of RNA was demonstrated by the absorbance ratio at 280 nm and 260 nm of 1.05 (A_280 nm_/A_260 nm_) (Figure S1C in Text S1). The MBP-N_Δ21_ monomer migrated as a single protein of about 100 kDa on a denaturing 4–20% gradient PAGE (Figure S1B in Text S1). Incubation of MBP-N_Δ21_ at 20°C overnight in the presence of P_60_ resulted in the displacement of the bacterial RNA from N, the dissociation of the oligomeric N-RNA complexes and the formation of an new species that eluted at 14.1 mL (Figure S2A in Text S1). The analysis by SEC-MALLS indicated a weight-averaged molecular mass of 92±2 kDa in agreement with the calculated molecular mass of the MBP-N_Δ21_^0^-P_60_ complex (calculated mass: 88,326 Da (MBP-N_Δ21_)+8,053 Da (P_60_) = 96,379 Da). The co-elution of MBP-N_Δ21_ and P_60_ was confirmed by denaturing 4–20% gradient PAGE (Figure S2B in Text S1). The complex contained much less RNA as shown by the absorbance spectrum (A_280 nm_/A_260 nm_ = 1.60) (Figure S2C in Text S1). After cleavage of the MBP tag with the TEV protease, the resulting N_Δ21_^0^-P_60_ complex was purified by Ni^2+^ chelate affinity chromatography followed by SEC. The complex of N_Δ21_ with full-length P dimer [11] was then prepared by incubating the purified N_Δ21_^0^-P_60_ complex overnight with the intact P dimer.

### Solution properties of the N_Δ21_^0^-P and N_Δ21_^0^-P_60_ complexes

**Figure 1:**
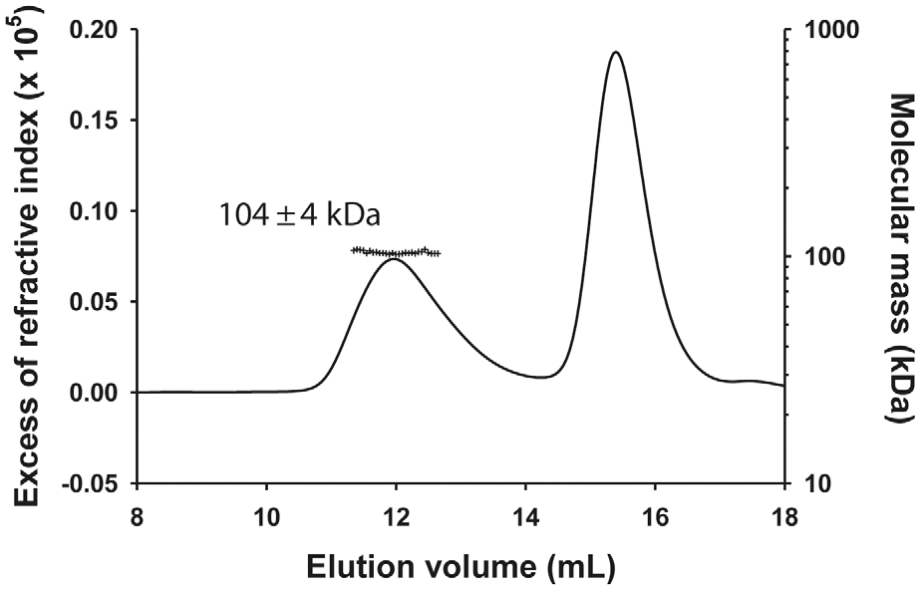
Heterotrimeric VSV N_Δ21_^0^-P dimer complex in solution. The complex formed between N_Δ21_ and full-length P dimer in solution was analyzed by SEC-MALLS. The N_Δ21_^0^-P complex elutes at 12.0 mL and the remaining N_Δ21_^0^-P_60_ complex elutes at 15.6 mL (line). The molecular mass of 104±4 kDa (crosses) indicates a 1∶2 complex between one RNA-free N_Δ21_ and two P molecules in accordance with the previous observation that P forms exclusively dimers in solution and with the N^0^-P_2_ complex determined for rabies virus.

The molecular mass of the N_Δ21_^0^-P dimer complex determined by SEC-MALLS was constant throughout the chromatographic peak indicating that the complex was monodisperse (M_w_/M_n_ = 1.00±0.01), and the molecular mass of 104±4 kDa was consistent with that of a heterotrimer composed of one N_Δ21_ and an intact P dimer (calculated mass: 45,377 Da (N_Δ21_)+2×30,976 Da = 107,329 Da) (Figure 1) in accordance with the dimeric state of P in solution [11] and with a previous determination for the rabies virus N^0^-P complex by native mass spectrometry [17]. The hydrodynamic radius (R_S_) of 5.8±0.1 nm is about 1.5 fold larger than that for a globular particle of the same molecular mass (calculated R_S_ = 4.0 nm) reflecting the elongated shape of the complex and the existence of a long N-terminal disordered region (aa 1–106) [11], [12].

**Figure 2:**
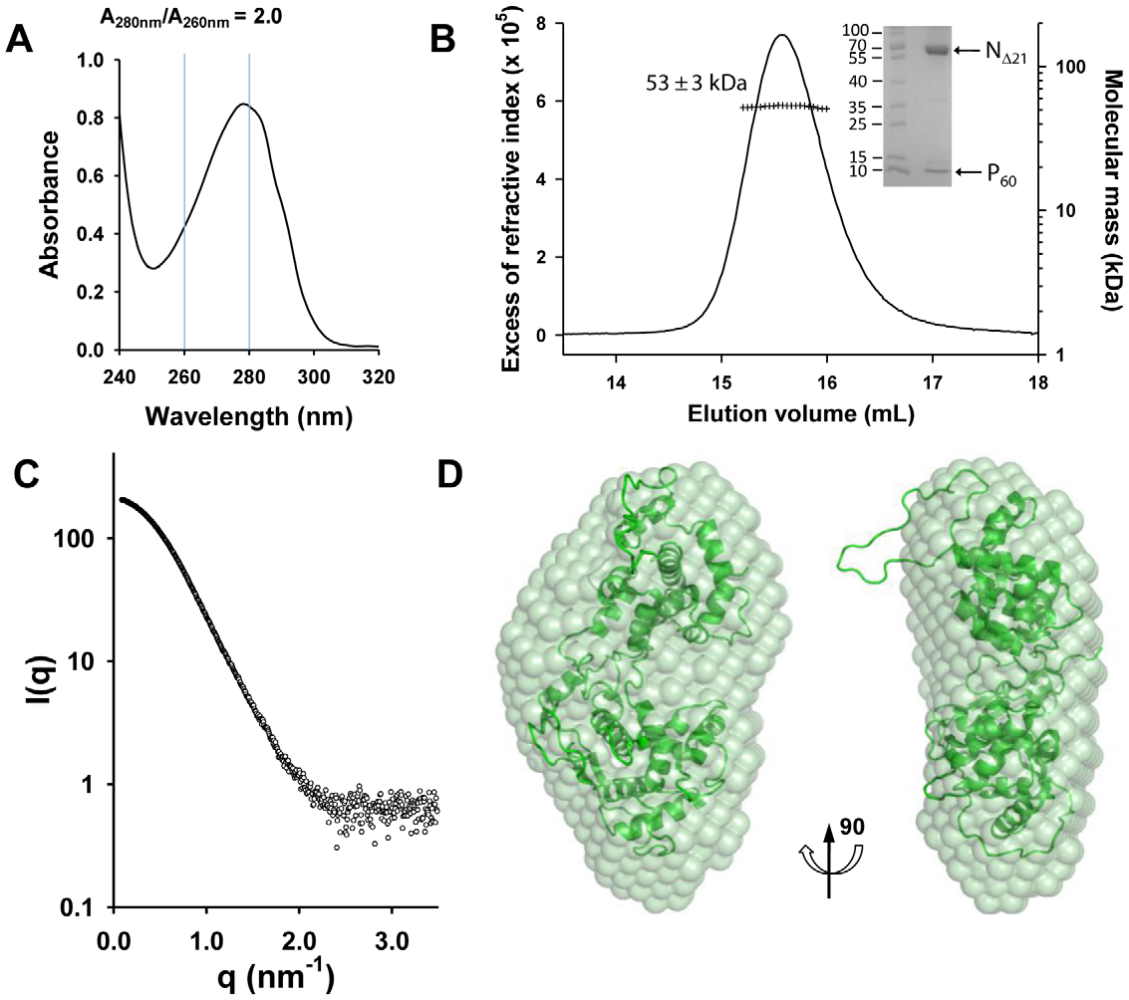
Heterodimeric VSV N_Δ21_^0^-P_60_ complex in solution. (A) The absence of RNA in the complex is revealed by the UV absorbance spectrum, which exhibits an A_280 nm_/A_260 nm_ ratio of 2.0. (B) Analysis of the N_Δ21_^0^-P_60_ complex by SEC-MALLS. The complex elutes as a single peak at 15.6 mL (line), and the presence of the two proteins in the complex is demonstrated by SDS-PAGE analysis of the peak fraction using Coomassie blue staining (inset). The molecular mass of 53±2 kDa (crosses) indicates a 1∶1 complex between N_Δ21_ and P_60_ (calculated molecular mass = 45,377 Da (N_Δ21_)+8,053 Da (P_60_) = 53,430 Da). (C) Experimental SAXS data (open circles) up to 3.5 nm^−1^. The SAXS curve recorded at ESRF beamline ID 14-3 shows the scattering intensity I(q) as a function of the scattering vector, 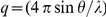. (D) Average *ab initio* bead model of the N_Δ21_^0^-P_60_ complex. The N protomer extracted from the circular N-RNA complex (2GIC chain E) fits to the SAXS-derived model, except for the N_CT_-loop.

The N_Δ21_^0^-P_60_ complex contained no RNA (Figure 2A), and its R_S_ of 3.2±0.1 nm and molecular mass of 53±3 kDa indicated a globular 1∶1 complex (Figure 2B), which agrees with the fact that P_60_ does not contain the dimerization domain of P [12], [18]. The radius of gyration (R_g_) of the N_Δ21_^0^-P_60_ complex determined from SAXS data (2.7±0.1 nm) (Figure S3 and Table S1 in Text S1) was similar to that of a single N protomer extracted from the N-RNA complex (2.8 nm) [15], but the calculated curve of the extracted protein poorly fitted the experimental curve of N_Δ21_^0^-P_60_, probably because of the presence of P_60_ (Figures S4A and S4B in Text S1). *Ab initio* bead models reconstructed from SAXS data [19] (Figure 2C) could easily accommodate the structure of an isolated N protomer deleted of its N_NT_-arm, except for the N_CT_-loop, which is likely to adopt a different conformation in solution (Figure 2D). Although the low resolution of the model precluded the precise localization of P_60_, the absence of an empty groove at the interface between N_NTD_ and N_CTD_ suggests that P_60_ could bind in this region. These results clearly show that P_60_ fulfils both chaperone functions of P in maintaining N^0^ soluble and free of RNA, and because the size and flexibility of full-length P render the N_Δ21_^0^-P complex unsuitable for X-ray crystallography and NMR studies, the N_Δ21_^0^-P_60_ complex was used for further structural characterization.

### Crystal structure of a decameric N_Δ21_^0^-P_60_ complex

**Figure 3:**
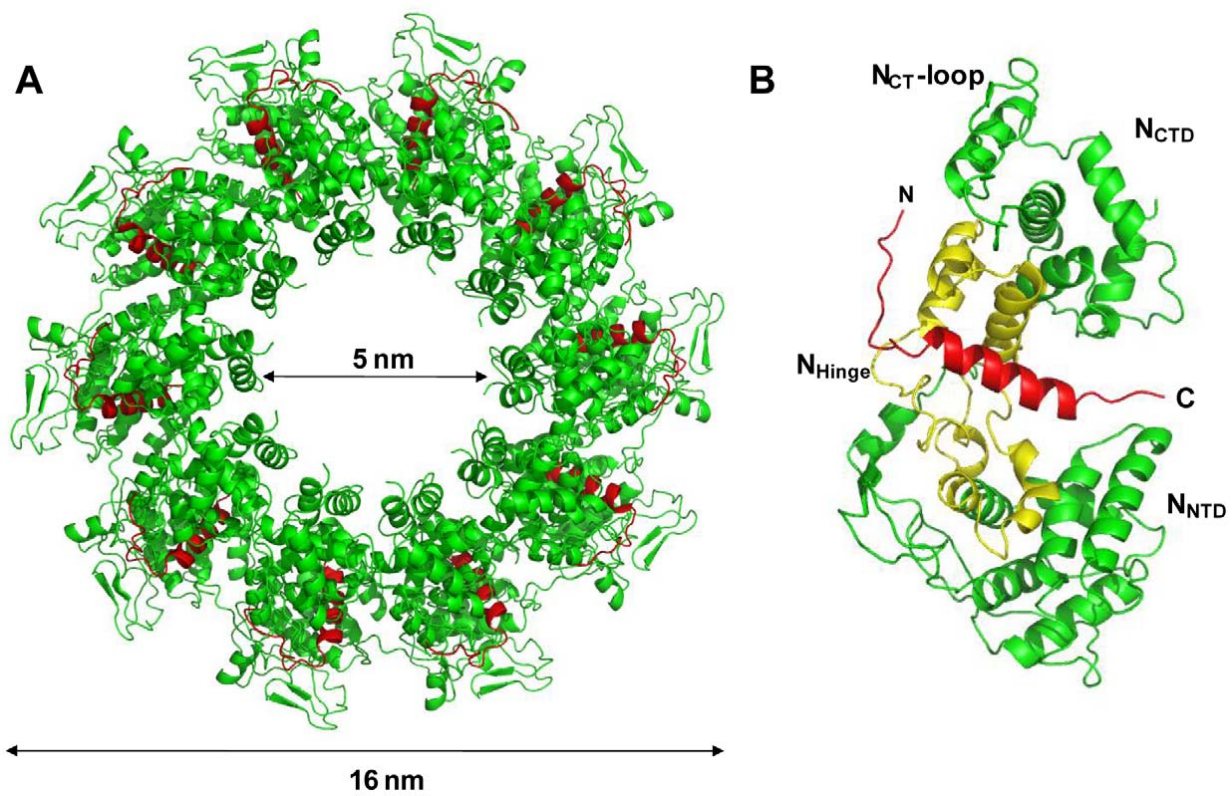
Crystal structure of a decameric form of the N_Δ21_^0^-P_60_ complex. (A) Overall structure of the decamer of N_Δ21_^0^-P_60_ complex. N_Δ21_ is shown in green and P_60_ in red. (B) Ribbon representation of one protomer of N_Δ21_^0^-P_60_. The central hinge region of N (aa 200–300) is shown in yellow and P_60_ (aa 6–35) is shown in red.

The N_Δ21_^0^-P_60_ complex crystallized at low pH as a decameric circular complex. The structure was solved at a resolution of 3.0 Å by molecular replacement using the structure of an N protomer derived from the N-RNA complex [15] (Table 1). The structure of N in the N_Δ21_^0^-P_60_ complex was almost identical to that of N in the N-RNA complex (rmsd = 0.96 Å) (Figure 3A and Figure S5 in Text S1) [15]. The complex contained no RNA, but instead, in each protomer, residues 6 to 35 of P_60_ were visible in a groove formed by residues of the hinge region of N (aa 200–300) at the junction between N_NTD_ and N_CTD_ (Figure 3B). Previous observations showed that the isolated N^0^-binding region of P transiently populates α-helical conformers in the region 2–12 and 25–31 [13], and that residues 11 to 30 of P are essential for forming the N^0^-P complex [7]. Upon binding to N_Δ21_, the second fluctuating helix is stabilized and extends from amino acids 17 to 31 (Figure 3B). The theoretical SAXS curve calculated from the crystal structure of one protomer of the N_Δ21_^0^-P_60_ complex fits adequately the experimental curve of the soluble complex (Figure S4A and S4C in Text S1), and the structure is perfectly accommodated within the *ab initio* bead model (Figure S4D in Text S1) showing that the structure of the N_Δ21_^0^-P_60_ complex in solution is the same as that in the crystal.

**Table 1:** Data collection and refinement statistics (molecular replacement).

	Crystal 1
**Data collection**	
Space group	P2_1_2_1_2
Cell dimensions	
*a*, *b*, *c* (Å)	74.56, 171.97, 239.86
α, β, γ (°)	90.0, 90.0, 90.0
Resolution (Å)^a^	59.97-3.03 (3.20-3.03)
*R*_sym_ a	11.4%(28.1%)
*I*/σ*I* a	9(3.5)
Completeness (%)^a^	89.9 (80.7)
Redundancy	3.1 (2.5)
**Refinement**	
Resolution (Å)	59.97-3.03 (3.11-3.03)
No. reflections	168,372
*R*_work_/*R*_free_	24.4%/27.7%
No. atoms	
Protein	17,105
Water	455
R.m.s. deviations	
Bond lengths (Å)	0.006
Bond angles (°)	0.893
Ramachandran favored	92.6%
Ramachandran allowed	98.4%

a Values in parentheses are for highest-resolution shell.

**Figure 4:**
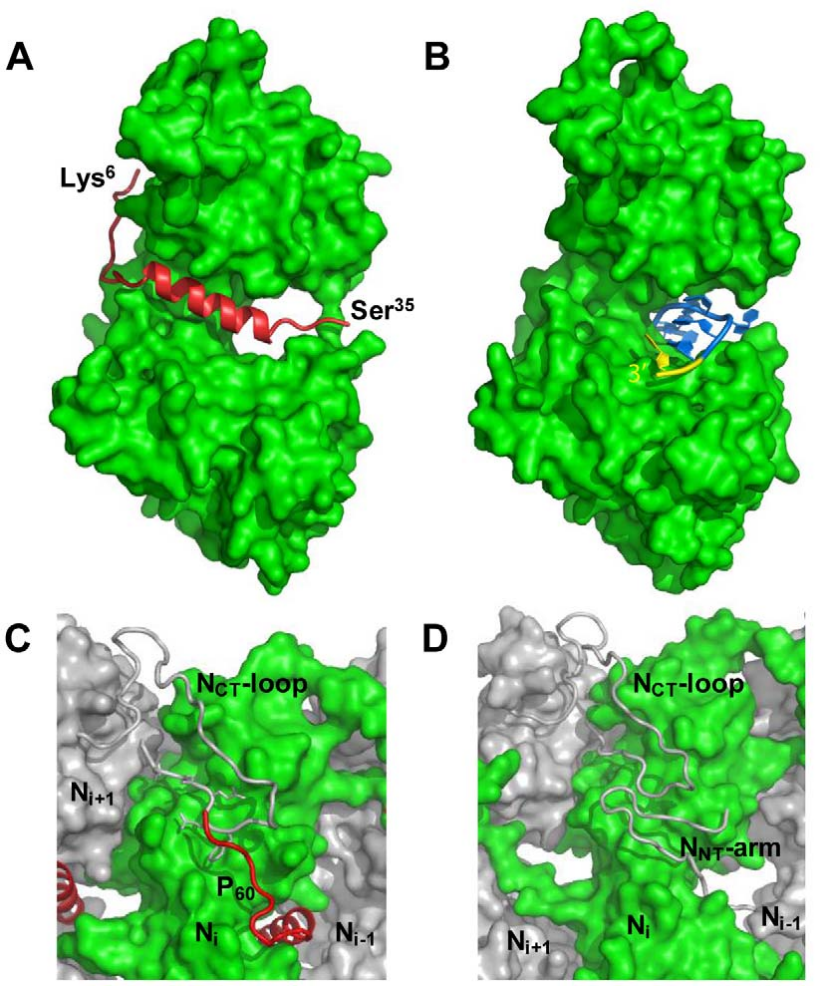
P binding hinders RNA binding and self-assembly of soluble N. (A) Representations of one protomer from the N_Δ21_^0^-P_60_ complex. The N protomer is shown as space filling model in green and P_60_ is shown as a cartoon representation in red. The visible N- and C-terminal residues of P_60_ are labeled. (B) Representations of one protomer from the w.t. N-RNA complex. The 3′ terminal nucleotide of the RNA molecule is shown in yellow. These representations show that P_60_ fills the RNA-binding cavity on the side of N that accommodates the 3′-end of the RNA molecule. (C, D) Close-up of the interactions between exchangeable sub-domains in the circular N_Δ21_^0^-P_60_ and N-RNA complexes. In the w.t. N-RNA complex (D), the N_NT_-arm of protomer N_i−1_ contacts the N_CT_-loop of protomer N_i+1_ while both sub-domains are docked on the back-side of protomer N_i_ (in green). In the N_Δ21_^0^-P_60_ complex (C), the N-terminal extremity of P_60_ docks on protomer N_i_ (in green) at the position of the N_NT_-arm of protomer N_i−1_ and contacts the N_CT_-loop of protomer N_i+1_. These representations suggest that P_60_ interferes with the assembly of N in the absence of RNA.

The structure of the N_Δ21_^0^-P_60_ complex shows how the N^0^-binding MoRE of P prevents both the interaction with RNA and the self-assembly of soluble RNA-free N. The binding site of P is different from that of RNA but both sites do overlap (Figures 4A, 4B and Figure S5 in Text S1), and the C-terminal turn of the α-helix of P_60_ (aa 27–31) together with the following residues (aa 32–35) block the RNA binding cavity (Figure 4A) and inhibit RNA binding. Concomitantly, the other extremity of the MoRE (aa 6–13) docks in a shallow groove on the backside of the N protomer which, in the multimeric N-RNA complex, is occupied by the N_NT_-arm of the adjacent N_i−1_ protomer and whose bottom is made up of the N_CT_-loop of the N_i+1_ protomer (Figure 4C and 4D). With w.t. N, the N-terminal part of the MoRE of P will compete with the N_NT_-arm of a neighboring N molecule and therefore interferes with the polymerization of N in the absence of RNA.

**Figure 5:**
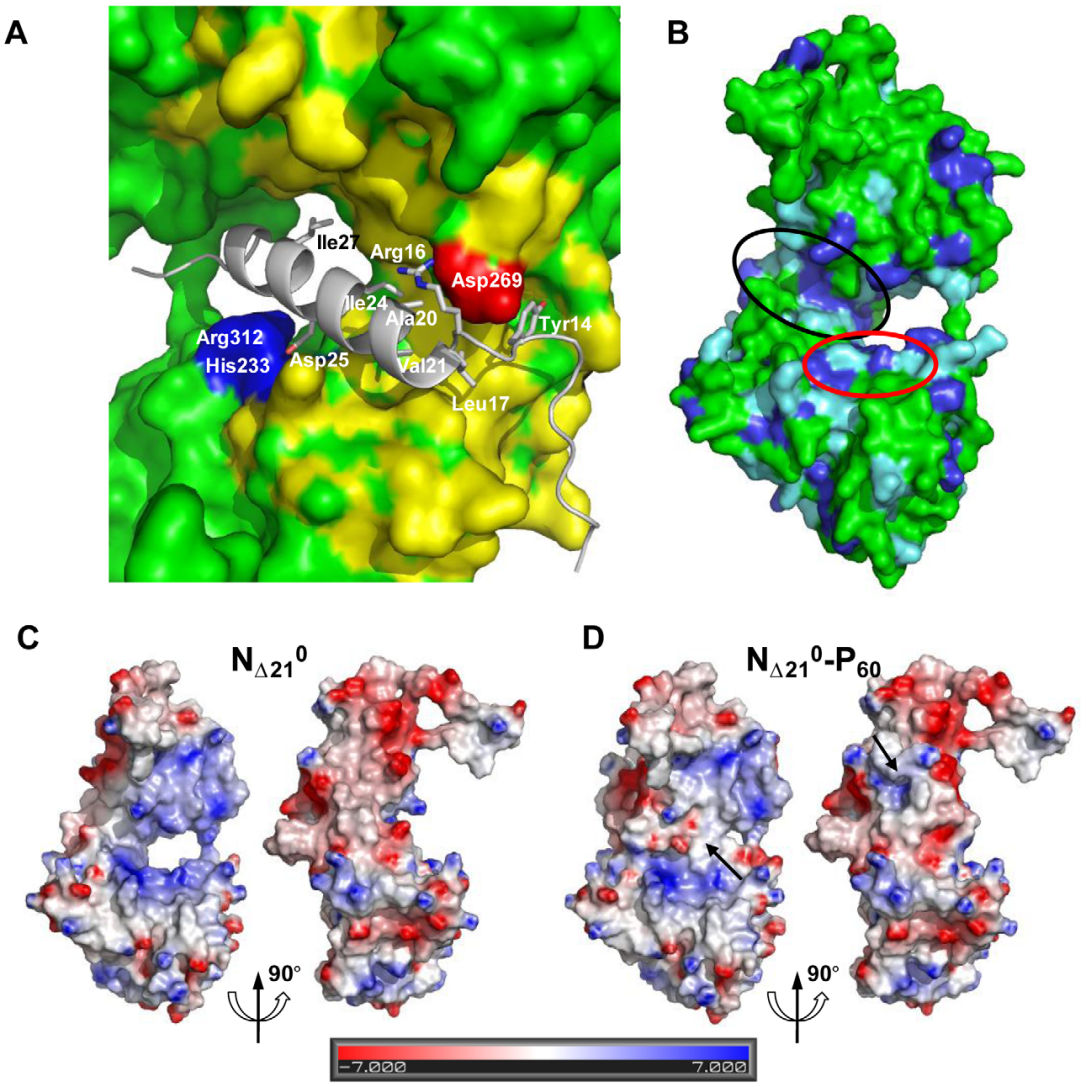
Surface properties and amino acid conservation in the P binding site. (A) Close up of the interface between RNA-free N_Δ21_ and P_60_ showing the hydrophobic contacts and salt bridges. Residues 17 to 31 of P_60_ fold into an amphipathic α-helix that lies in a hydrophobic cavity formed by residues of the hinge region of N and is stabilized by hydrophobic contacts involving residues of P_60_ spaced i+3 or i+4 (Leu^17^, Val^21^, Ile^24^ and Ile^27^). Tyr^14^ docks into a small cavity lined with hydrophobic residues. Hydrophobic side chains in N are colored in yellow and hydrophobic side chains of P_60_ are labeled. The complex is also stabilized by salt bridges between Asp^25^ of P_60_ and Arg^312^ and His^233^ of N (in blue) and between Arg^16^ of P_60_ and Asp^269^ of N (in red). (B) Amino acid sequence conservation between VSV and RAV N. Identical residues are shown in dark blue and similar residues are shown in light blue. The surface area circled in black shows the binding groove of P and the surface area circle in red shows the hydrophobic site common to both P and the bases at the 3′ end of the RNA. (C, D) Electrostatic surface potential of the N_Δ21_^0^ protein (C) compared with that of the N_Δ21_^0^-P_60_ complex (D). Both panels show in the same orientations the two sides of the N_Δ21_^0^ protein involved in binding the MoRE of P. The arrows indicate regions in which the electrostatic surface potential of N is modified by the presence of the peptide. The surface potentials were calculated with the Delphi program and are color-coded on the surface from red (negatively charged residues, −7 kcal/mol) to blue (positively charged residues, +7 kcal/mol).

The MoRE of VSV P (aa 6–35) binds to the central hinge region of N mainly through hydrophobic interactions. The amphipathic α-helix of P, together with residues 14 to 16, inserts into a hydrophobic groove of N. Tyr14 perfectly fits into a small hydrophobic pocket (Figure 5A). In addition, the complex is stabilized by three intermolecular salt bridges (Figure 5A). This region of N (aa 200–300), which also plays a central role in binding RNA is highly conserved among VSV serotypes as well as in rabies virus (RAV) [20], [21] (Figure S6 in Text S1). The hinge region of VSV N exhibits 30% identity in amino acid sequence with that of RAV N, as compared with 21% and 13% for the N- and C-terminal lobes, respectively. Figure 5B shows that several hydrophobic residues lining the binding groove of P are conserved between VSV and RAV N, as well as Arg^312^, suggesting that a similar complex forms in RAV (Figure S6 in Text S1). In the N-RNA complex, the RNA molecule interacts with N through electrostatic interactions between phosphate groups of the RNA backbone and basic residues of the protein, while the bases of three nucleotides (nt. 5, 7 and 8) are docked onto an hydrophobic surface of the RNA binding groove [15], which is also part of the MoRE binding site (Figure 5B). However, none of the basic residues of VSV N directly contacting the RNA backbone in the N-RNA complex is involved in the interaction with the MoRE of P. A similar mode of RNA binding was observed in the RAV N-RNA complex [14], but only two arginines out of the six residues involved in direct interactions with phosphate groups in the VSV complex are conserved in the RAV complex [20].

The RNA binding groove of N is rich in basic residues forming a highly positive surface area (Figure 5C), while the backside of N_CTD_ harbors a negative surface potential (Figure 5C). The MoRE of P (aa 6–35) has a bipolar distribution of charges with a positive pole at its N-terminus and a negative pole its C-terminus. In the N_Δ21_^0^-P_60_ complex, the negative pole of P localizes in the RNA binding groove, while the positive pole docks on the backside of N_CTD_, modifying the distribution of electrostatic potentials on these surfaces of N and suggesting that electrostatics could play a role in orientating P before binding (Figures 5C and 5D). The crystallization is at pH 4.6 and it is likely that protonation of acidic groups reduces repulsion forces that keep the N_Δ21_^0^-P_60_ complex in its isolated form at pH 7.0.

### NMR spectroscopy

**Figure 6:**
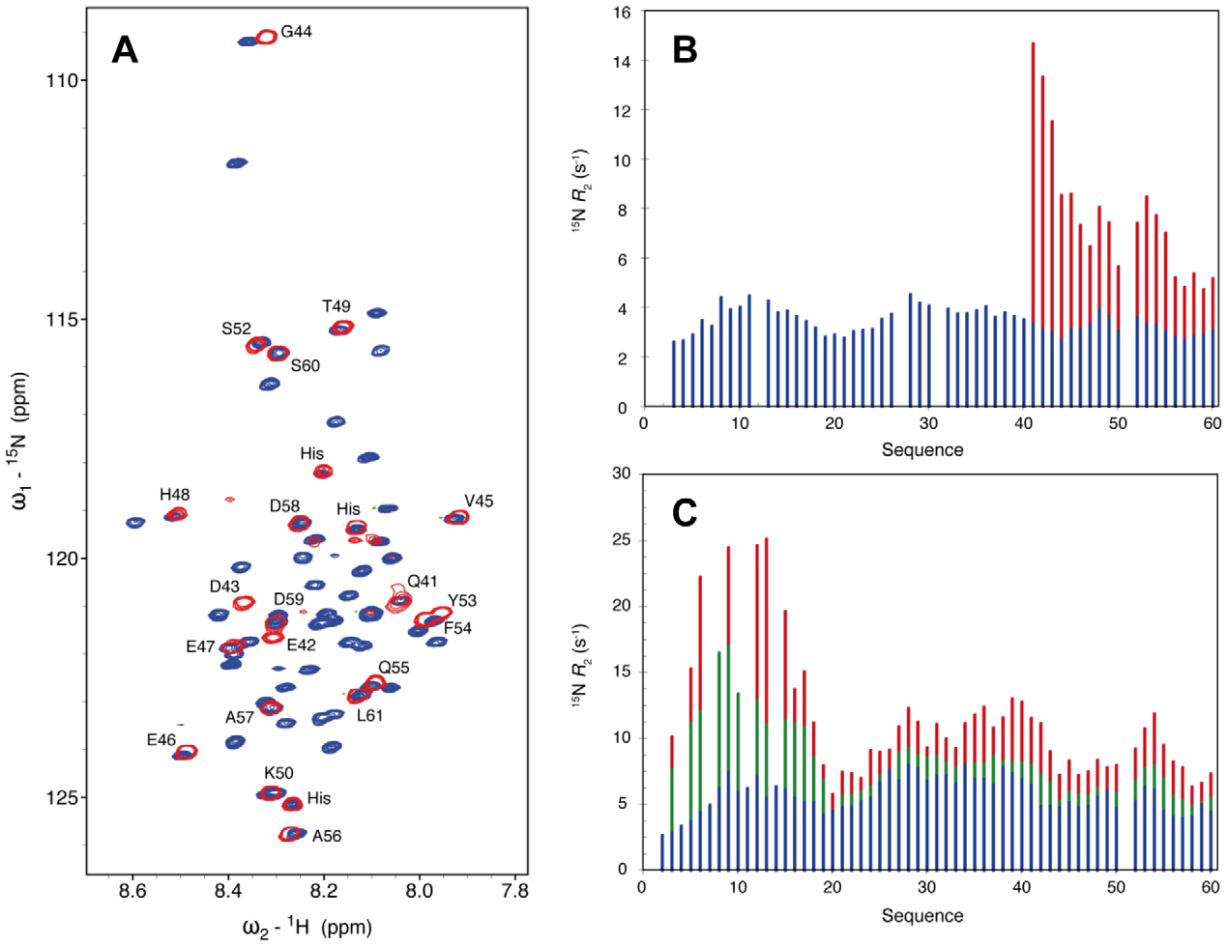
The N-terminal and C-terminal region of P_60_ flanking the MoRE exhibits conformational flexibility in the soluble complex. (A) Comparison of the 2D ^1^H-^15^N HSQC NMR spectra of free ^15^N, ^13^C, ^2^H-labelled P_60_ (blue) and in complex with N_Δ21_ (red). Both spectra were recorded at 14.1 T and 25°C in 20 mM Tris-HCl, 150 mM NaCl, 50 mM Glu, 50 mM Arg with 10% D_2_O adjusted to pH 6.0. The labels indicate the assignment of the resonances of the complex. (B) ^15^N *R*_2_ spin relaxation rates measured under the same conditions as (A) for the free ^15^N-labeled P_60_ (blue) and the N_Δ21_^0^-P_60_ complex (red). (C) ^15^N *R*_2_ spin relaxation rates measured at 14.1 T and 10°C of the free ^15^N-labeled P_60_ (blue), a mixture of 0.27 mM ^15^N-labeled P_60_ and 0.09 mM unlabeled MBP-N_Δ21_ (green) and a mixture of 0.24 mM ^15^N-labeled P_60_ and 0.17 mM unlabeled MBP-N_Δ21_ (red).

In the crystal structure of the N_Δ21_^0^-P_60_ complex residues 6 to 13 and 32 to 35 of P_60_ exhibit conformational heterogeneity (Figure S7 in Text S1), while residues 1 to 5 and 36 to 68 are not visible. To further characterize the conformational dynamics of these parts of P_60_ in the soluble N_Δ21_^0^-P_60_ complex, we used nuclear magnetic resonance (NMR) spectroscopy. Initially, spectra of ^15^N, ^13^C, ^2^H-labeled P_60_ in complex with unlabeled N_Δ21_ were recorded. In a complex of this size (53 kDa) NMR signals are significantly broadened, precluding their detection, but in the HSQC spectrum of the N_Δ21_^0^-P_60_ complex, resonances corresponding to the last 28 amino acids of P_60_ (aa 41–60+linker+His_6_ tag) are clearly visible (Figure 6A), suggesting that this tail remains free and flexible in the complex. Comparison with the free peptide showed that most resonances superimpose. Small chemical shift differences were observed for residues Q^41^ to G^44^, probably due to the proximity of the bulk complex, and for two aromatic residues (Y^53^, F^54^) suggesting weak interactions of these residues with N. The amide backbone ^15^N transverse relaxation rate constant (*R_2_*) is sensitive to rapid fluctuations at the pico- to nanosecond time scale, as well as to chemical shift exchange on the micro- to millisecond time scale. Transverse relaxation of the visible resonances increased from the C-terminus to the region containing the bound helix, indicating a corresponding increase in rigidity of the backbone (Figure 6B). Very weak additional peaks up-field shifted in the amide proton dimension were detected in a ^15^N-^1^H TROSY spectrum further supporting folding of the helical element upon binding (data not shown).

In a second experiment, addition of sub-stoichiometric amounts of unlabeled N_Δ21_ attached to MBP (to maintain solubility) to ^15^N-labeled P_60_ resulted in an overall reduction in the intensity of the peaks in the HSQC spectrum, in proportion of the amount of added MBP-N_Δ21_ indicating that a fraction of P_60_ formed a complex with MBP-N_Δ21_. Spin relaxation measurements of equilibrium mixtures of free and bound peptide refine our understanding of the dynamics of the system. Systematically larger *R_2_* values observed for residues 1 to 17 reveal additional contributions from chemical shift exchange (*R_ex_*) that are not present in the free form of the peptide and that increase significantly upon increasing the molar ratio of MBP-N_Δ21_ to P_60_ (Figure 5C). This indicates that the N-terminal part of P_60_ experiences conformational exchange when in complex with N_Δ21_ with an interconversion rate on the micro to millisecond timescale. In the crystal structure, residues 6 to 13 bind in place of the N_NT_-arm of the protomer N_i−1_ and pack onto the N_CT_-loop of protomer N_i+1_ (Figures 4C and 4D), but in the isolated form of the complex, the N_CT_-loop binding surface for these residues is missing. These results confirmed that in the solution, like in the crystal, residues 17 to 35 of P form a stable complex with N, while the flanking N- and C-terminal regions remain dynamic. The flanking C-terminal part (aa 40–60) behaves as a flexible tail and shows little evidence of interaction with N. The flanking N-terminal region (aa 1–16) interacts with N but undergoes conformational exchange. These flexible regions seem dispensable for the chaperone activities of P since a shorter peptide encompassing residues 7 to 40 of P is also capable of displacing bacterial RNA from N_Δ21_ and of forming a soluble heterodimeric 1∶1 complex with this protein (data not shown).

## Discussion

In the current study, we have determined the structure of the N^0^-binding MoRE of P bound to N and demonstrated that the regions of P flanking the MoRE conserve some flexibility in the N^0^-P complex. Because the N^0^-P complex is required for the replication of the virus [5], [22], inhibition of its formation might represent an interesting target for blocking viral replication and could explain the recent observations that a homologous peptide (P_60_) from rabies virus P inhibits viral replication [23].

### The N_Δ21_^0^-P_60_ complex as a model of the N^0^-P complex

Our results demonstrate that the reconstituted N_Δ21_^0^-P_60_ complex is a suitable model for the viral N^0^-P complex in agreement with previous studies. Both the crystal structure and the NMR spectroscopy experiments show that the MoRE of P, which adopts a stable conformation upon binding to N, includes residues 6 to 35 and corresponds closely to the fragment that was previously identified as essential and sufficient for maintaining N in a soluble form (aa 11–30) [7].

Previous studies revealed that VSV P is a dimeric and modular protein in which the N-terminal part (aa 1–106) is globally disordered [11], [12], [13], [18], [24], [25]. The dimerization domain of P is localized in the central region of the protein (aa 107–177) [18] and is therefore not present in P_60_, which is monomeric [13]. The stoichiometry of the N_Δ21_^0^-P_60_ complex (1∶1) shows that one MoRE of P is capable of binding one N molecule. The stoichiometry of the complex formed between N_Δ21_ and full-length P (1∶2) in the concentration range used here suggests that a single N_Δ21_ is bound to P dimer. The remaining part of the P dimer is tethered to N_Δ21_ through a flexible linker, in agreement with the large hydrodynamic radius measured here for the N_Δ21_^0^-P dimer complex. In isolation, the N-terminal region of P contains two transient α helices (aa 2–12 and 25–38) [13]. In the crystal structure, the second helix is stabilized and extends from residue 17 to residue 35, whereas the first helix is not present. Residues 1 to 5 are not visible and residues 6 to 13 adopt different conformations in the different protomers of the circular complex. Because NMR spectroscopy reveals that this N-terminal region of P (aa 1–17) is in chemical shift exchange, it is possible that in solution it adopts different conformations bound in different orientations on the surface of N, but that only those docked into the backside groove of N allow the packing of the N_Δ21_^0^-P_60_ complex into crystals and were thus selected during the crystallization process.

The truncated form of N (N_Δ21_) conserves the ability of self-association in the presence of RNA and, like w.t. N, forms oligomeric N-RNA complexes when expressed in bacteria. As assumed from the structure of the oligomeric N-RNA complex [15], the N_NT_-arm stabilizes the multimeric N-RNA complexes by linking together adjacent N protomers. The N-RNA complex formed with w.t. N could not be dissociated by the addition of full-length P or of a fragment of P encompassing the N^0^-binding region. However, the deletion of the N-terminal sub-domain destabilized the complex and allowed P_60_ to displace the RNA molecule and disassemble the multimeric N-RNA complex. In a previous study, the co-expression of P with a similar variant of VSV N lacking the first 22 amino acids (N_Δ22_) led to the production of complexes of different sizes containing N_Δ22_ and P but not of N-RNA complexes, suggesting a role for the N-terminal region of N in the encapsidation of the RNA [10]. Assuming an equilibrium between the N^0^-P complex and the multimeric N-RNA complex, with w.t. N, the stabilization brought by the N_NT_-arm to the multimeric assembly would displace the equilibrium towards the formation of the N-RNA complex. In the absence of the N_NT_-arm, the truncated N molecules assemble onto cellular RNAs as seen in our expression system, but in the presence of co-expressed P, like upon addition of P_60_ to our purified MBP-N_Δ21_-RNA complexes, the equilibrium is displaced towards the formation of the N^0^-P complexes. The absence of N-RNA complex in cells co-expressing N_Δ22_ and P may not result from a default of encapsidation but rather from the displacement of the equilibrium towards N^0^-P.

Unexpectedly, the N_Δ21_^0^-P_60_ complex failed to crystallize as a heterodimer but crystallized into circular decamers of heterodimers. With the exception of the missing N_NT_-arm, the structure of N_Δ21_ in the N_Δ21_^0^-P_60_ complex is very similar to that of N in the decameric N-RNA complex, with less than 1 Å r.m.s.d. between the two structures. Different explanations why multimerization occurs under crystallization conditions can be proposed. Firstly, the N_Δ21_^0^-P_60_ complex crystallized at pH 4.6 like VSV circular N-RNA complexes [15], while solution SEC-MALLS and SAXS experiments were performed at pH 7.5 and NMR experiments at pH 6.0. A modification of the electrostatic surface potential (Figures 5C and 5D) could affect the equilibrium between heterodimeric and multimeric N_Δ21_^0^-P_60_ complex. No evidence of multimerization was, however, found in solution at pH 4.6 in the concentration range used for SEC-MALLS and SAXS experiments. Secondly, the ring-like structure of ten protomers appears as a favored organization of VSV N, likely reflecting on some geometrical and/or surface properties of the protein. VSV N forms ring-like structures mostly containing ten N subunits in the presence of non-specific RNA when expressed in a recombinant system [15], [26]. The RNA can be removed from these ring-like structures without disrupting the multimeric assembly [27]. A single amino acid variant of VSV N that is no longer capable of binding RNA also crystallized into a decameric assembly of empty N molecules [10]. These circular N-RNA complexes are artifacts of the crystallization process because the actual nucleocapsid is very long and cannot form rings. However, in the virion, the nucleocapsid adopts a bullet-shaped structure composed of a trunk in which the nucleocapsid regularly spirals into superposed turns of 37.5 subunits of N and of a tip which is formed of seven turns containing varying numbers of subunits [1]. The upper turn of the bullet tip, which may represent the nucleation centre from which the particle assembles, resembles a decameric ring, suggesting that the assembly in ten members ring or spiral corresponds to an optimal side-by-side orientation between adjacent N subunits. The RNA-free N_Δ21_^0^-P_60_ complex is capable of assembling into circular multimers, and it seems likely that an increase of the concentration of the N_Δ21_^0^-P_60_ complex under the crystallization conditions together with a change in pH shift the equilibrium towards the multimers.

This raises the question of the effect of crystallization on the structure of the N_Δ21_^0^-P_60_ complex. The SAXS curve calculated for N_Δ21_^0^-P_60_ protomer extracted from the crystal structure perfectly reproduced the experimental curve of the soluble complex, while NMR spectroscopy clearly shows that the same segment of P (aa 17–35) is involved in a stable complex with N in solution and in the crystal, arguing that crystallization has no major effect on the structure of the more rigid part of the complex. In solution, the N-terminal part of the MoRE of P (aa 1–16) appears to be in conformational exchange and could thus exist in different conformers including those observed in the crystal in which residues 6 to 16 are docked onto the backside groove of N. Crystallization of the N_Δ21_^0^-P_60_ complex may thus select the more compact conformers and therefore not reproduce the conformational diversity of this region that is found in solution. In addition, the high conservation rate of residues of N forming the binding surface for the MoRE of P, both within VSV serotypes and between the evolutionarily more distant VSV and RAV, supports the localization of the interface between the two proteins and hints at the formation of a similar complex in RAV.

### Mechanisms of chaperone activities

The characterizations in solution indicate that the N_Δ21_^0^-P_60_ complex is RNA-free heterodimer and that, therefore, P_60_ or a shorter fragment of P (aa 7–40) fulfill both chaperone activities of P. The crystal structure of the N_Δ21_^0^-P_60_ complex clearly shows how the N^0^-binding region of P inhibits RNA binding by filling the RNA-binding groove of N. In solution, this part of P also forms a stable complex with N as seen by NMR spectroscopy. The structure also suggests how P prevents the self-assembly of N in the absence of RNA. In the bound form observed in the crystal structure, the N-terminal extremity of the MoRE (aa 6–16) directly competes with the N_NT_ arm of a neighboring N molecule. Assuming this region of P fluctuates between bound and free forms in the soluble N^0^-P complex, the free form may act as an entropic bristle, thereby also preventing the oligomerization of N. With full-length P dimer, the flexibility and the bulkiness of the remainder of the protein may also contribute to this effect by masking the binding interfaces for RNAs or other N molecules. In addition, the MoRE of P exhibits a bipolar distribution of charges, with a positive pole at its N-terminal extremity and a negative pole at its C-terminal extremity. Binding of the MoRE of P modifies the electrostatic surface potential of N, notably reducing the positive surface potential on one side of the molecule, and may thereby affect the side-by-side interaction with another N molecule.

**Figure 7:**
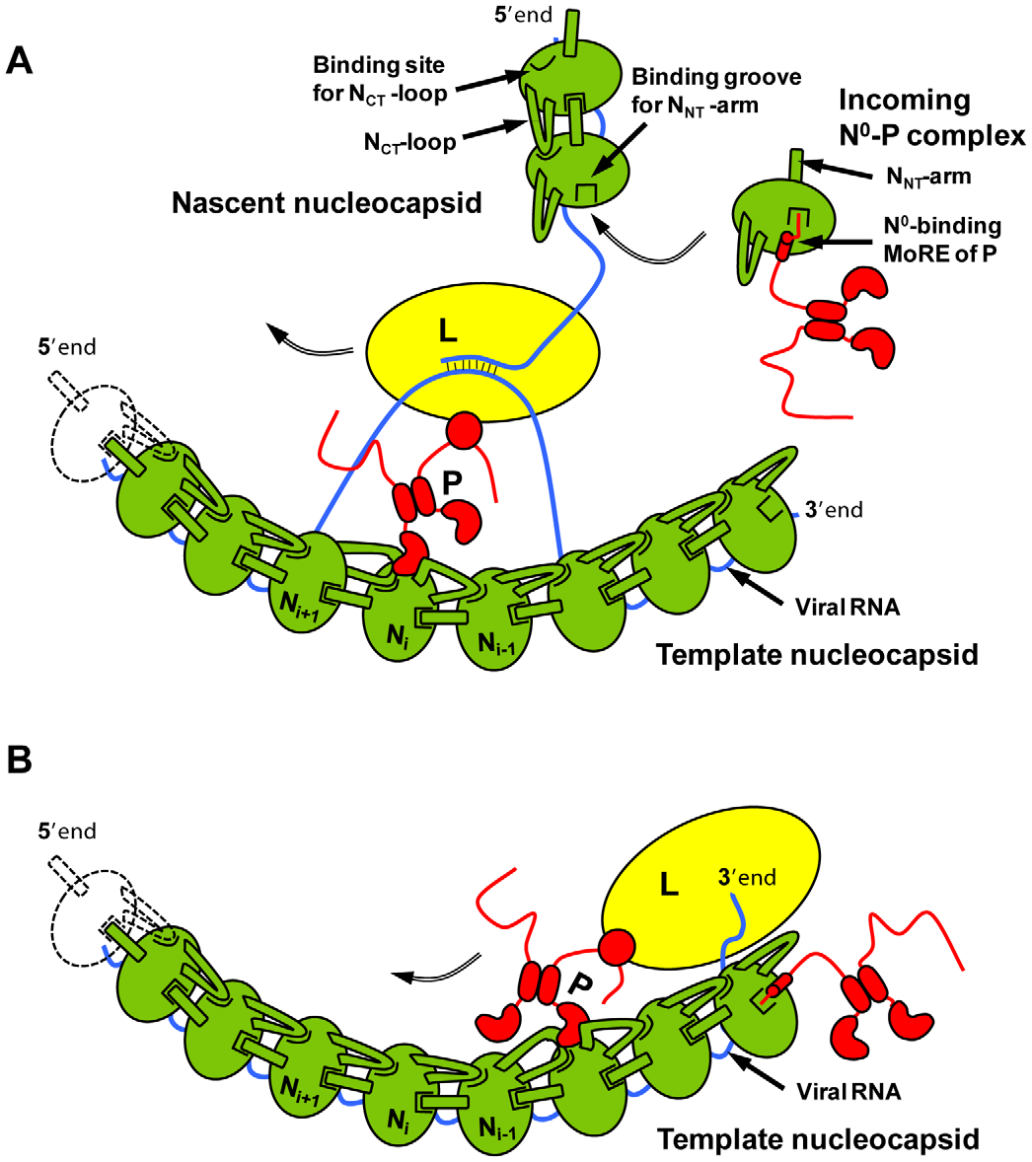
Schematic representations of the mechanism of RNA replication of VSV. The nucleoprotein (in green) forms with the RNA genome (blue line) the active template for the polymerase complex comprising the L (in yellow) and P (in red) proteins. (A) Encapsidation during RNA replication. During replication, the newly synthesized antigenomic or genomic RNA is encapsidated by nascent N molecules that are transferred from the soluble N^0^-P complex. In the N^0^-P complex, the N-terminal MoRE of P prevents host-cell RNA binding by obstructing the RNA binding groove and the self-assembly of N by interfering with the docking of the N_NT_-arm of another N. Upon the transfer of N to the growing viral RNA P is released, the binding groove for the N_NT_-arm is freed in the RNA-bound form and can accept the next incoming N molecule. (B) Initiation of RNA synthesis. By binding at the 3′ extremity of the nucleocapsid, the N-terminal MoRE of P might displace nucleotides from the N molecule and allow the polymerase to initiate RNA synthesis.

From the results presented here, we propose a hypothesis for the encapsidation of a newly synthesized RNA molecule during viral genome replication. By forming a complex with P, a nascent N molecule is prevented from binding to host-cell RNA and is preserved in a soluble form. During RNA replication, N is transferred to a growing RNA molecule and P is released. Little is known about the mechanism of this reaction or about the role played by the polymerase complex in this process. Our results show that the N_NT_-arm stabilizes the multimeric N-RNA complex and therefore suggest that the multimeric N-RNA complex is more stable than the N^0^-P complex. The transfer of N from the N^0^-P complex to the growing N-RNA complex could simply be driven by a higher stability of the N-RNA complex. Upon transfer of N onto the RNA and release of P, the backside groove of N of the last added N molecule is liberated and becomes available for accepting the N_NT_-arm of the next incoming N molecule (Figure 7A). By blocking the backside groove of N, the N-terminal part of P ensures that N molecules do not assemble into empty N polymers but assemble only onto an RNA molecule. It is also noteworthy that, in VSV, a high affinity binding site for the L protein was localized in the second half of the N-terminal disordered region of P [7], [28]. The dynamic nature of the N-terminal region of P and the proximity of the two binding sites may have significance for the mechanism of action of the transcription/replication machinery. The binding of N^0^ to P may prevent the simultaneous binding of L, or conversely, the simultaneous binding of N^0^ and L may modify the activity of the polymerase.

In addition to its role in RNA encapsidation, the binding of the N-terminal region of P to N may also provide a mechanism for the initiation of (+)RNA synthesis at the genome 3′ end and of (−)RNA synthesis at the antigenome 3′ end [29], [30]. Encapsidated RNA genome and antigenome are completely covered with the nucleoprotein and are not accessible to the RNA polymerase. However, the first N molecule at the 3′ extremity of nucleocapsids exposes its binding site for the N-terminal MoRE of P (Figure 7B). By binding to this surface, P may destabilize the N-RNA complex sufficiently to displace several nucleotides from the first N protomer and allow the polymerase access to the RNA.

## Materials and Methods

### Reconstitution of the N_Δ21_^0^-P and N_Δ21_^0^-P_60_ complexes

The cDNAs encoding vesicular stomatitis virus nucleoprotein or a fragment of this protein deleted of the 21 N-terminal residues were amplified by PCR and introduced into the pET-M40 plasmid (EMBL) using NcoI and XhoI restriction sites. The resulting constructs code for chimeric proteins that comprise an N-terminal maltose binding protein tag (MBP) and a tobacco etch virus (TEV) cleavage site. The cDNA encoding the 60 first N-terminal amino acids of VSV P (P_60_) was amplified by PCR and cloned into the pET28a plasmid containing a C-terminal His_6_-tag and a linker of two amino-acids (EL) using NcoI and XhoI restriction sites. All constructions were checked by DNA sequencing.

The plasmids were transformed into *Escherichia coli* Rosetta (DE3) cells and the expression of the recombinant proteins was induced with 1 mM isopropyl-1-thio-β-d-galactopyranoside (IPTG) for 18 h at 16°C. Cells were harvested by centrifugation, suspended in buffer A (20 mM Tris–HCl, pH 7.5, 150 mM NaCl, 5 mM DTT) containing protease inhibitors (Complete EDTA-Free, Roche Diagnostics) and disrupted by sonication. The extract was centrifuged at 20,000 g during 30 min at 4°C and the supernatant was filtered (0.45 µm). The MBP-fusion proteins were purified by affinity chromatography on amylose resin (New England Biolabs) followed by size exclusion chromatography (SEC) on a Superdex S200 column (GE Healthcare) equilibrated in buffer A. P_60_ was purified by affinity chromatography on a Ni^2+^ resin column (Quiagen) followed by SEC on a Superdex S75 column (GE Healthcare) equilibrated in buffer A supplemented with 50 mM Glu and 50 mM Arg. Samples for NMR spectroscopy were produced in M9 minimal medium containing MEM vitamins (Gibco). For producing ^15^N-labeled P_60_, the medium was supplemented with 1.0 g.L^−1^ of ^15^NH_4_Cl and 2.0 g.L^−1^ of unlabeled glucose, while for producing ^15^N, ^13^C, ^2^H-labeled P_60_ the minimal medium was prepared in D_2_O and supplemented with 1.0 g.L^−1^ of ^15^NH_4_Cl and 2.0 g.L^−1^ of ^13^C glucose.

The N_Δ21_^0^-P_60_ complex was prepared by incubating overnight at 4°C an excess of P_60_ with the MBP-N_Δ21_-RNA complexes. The MBP-N_Δ21_^0^-P_60_ complex was purified by Ni^2+^ chelate affinity chromatography by using the His-tag present on P_60_ to remove the excess of free MBP-N_Δ21_-RNA complex, followed by SEC on a Superdex S200 column equilibrated in buffer A and amylose affinity chromatography to eliminate unbound P_60_.

The MBP tag was removed by incubating the protein with the TEV protease overnight at 4°C. The N protein contains the additional N-terminal tripeptide GAM. The N_Δ21_^0^-P_60_ complex was then purified using a Ni^2+^ chelate affinity chromatography followed by SEC on a Superdex S200 column equilibrated in buffer A. This procedure yielded pure N_Δ21_^0^-P_60_ complex. The samples were checked by SDS-PAGE using denaturing 4–20% gradient PAGE (Biorad).

### Size exclusion chromatography (SEC) combined with detection by multi-angle laser light scattering (MALLS) and refractometry: SEC-MALLS

SEC was performed with a Superdex S200 column (GE Healthcare) equilibrated in 20 mM Tris–HCl, pH 7.5, 150 mM NaCl. Separations were performed at 20°C with a flow rate of 0.5 ml.min^−1^. 50 µL of a protein solution at a concentration ranging from 2.7 to 8.0 mg.mL^−1^ were injected. On-line multi-angle laser light scattering (MALLS) detection was performed with a DAWN-EOS detector (Wyatt Technology Corp., Santa Barbara, CA) using a laser emitting at 690 nm. Protein concentration was measured on-line by refractive index measurements using a RI2000 detector (Schambeck SFD) and a refractive index increment dn/dc = 0.185 mL.g^−1^. Data were analyzed and weight-averaged molecular masses (M_w_) were calculated using the software ASTRA V (Wyatt Technology Corp., Santa Barbara, CA) as described previously [11]. For size determination, the column was calibrated with proteins of known Stokes' radius (R_S_) [31].

### Small angle X-ray scattering (SAXS) and *ab initio* modeling

SAXS data were collected at the European Synchrotron Radiation Facility (E.S.R.F., Grenoble, France) on beamline ID14-3. The sample-to-detector distance was 1 m and the wavelength of the X-rays was 0.931 Å. Samples were contained in a 1.9 mm wide quartz capillary. The exposition time was optimized for reducing radiation damage. Data acquisition was performed at 20°C. Data reduction was performed using the established procedure available at ID14-3 and buffer background runs were subtracted from sample runs.

The SAXS profile of the N_Δ21_^0^-P_60_ complex was recorded for scattering vectors, 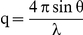, in the range 0.05 nm^−1^<q<3.5 nm^−1^. The profiles obtained at three different protein concentrations 2.7–8.0 mg.mL^−1^ had the same shape and were flat at low q values indicating the absence of significant aggregation (Figure S4A). The radius of gyration and forward intensity at zero angle (I(0)) were determined with the programs PRIMUS [32] by using the Guinier approximation at low q values (Figure S4B), in a 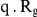 range up to 1.3:
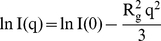
(1)The forward scattering intensity was calibrated using bovine serum albumin and lysozyme as references. The radius of gyration, R_g_, and the pair distance distribution function, P(r), were calculated with the program GNOM [33] (Figure S4C). The maximum dimension (D_max_) value was adjusted so that the R_g_ value obtained from GNOM agreed with that obtained from the Guinier analysis. The R_g_ values showed no significant dependence on protein concentration, confirming the absence of aggregation or intermolecular interactions in the concentration range used in this study. The measured R_g_ value of 2.7±0.1 nm (Table S1 in Text S1), and the molecular mass of 65±15 kDa derived from the scattering intensity at zero angle, I_0_, were in agreement with SEC-MALLS results. Additionally, the distance distribution function (Figure S4C) and the Kratky plot (Figure S4D) were typical of globular, folded proteins.

*Ab initio* low-resolution bead model reconstructions of the N_Δ21_^0^-P_60_ complex were performed from the scattering curves using the program DAMMIF [19]. This program restores a low-resolution shape of the protein as a volume filled with densely packed spheres (dummy atoms) that reproduces the experimental scattering curve by a simulated annealing minimization procedure. DAMMIF minimizes the interfacial area between the molecule and the solvent by imposing compactness and connectivity constraints. 20 independent models were generated with DAMMIF with no symmetry restriction and were combined with the program DAMAVER [34] yielding an average model that exhibited the common structural features of all reconstructions. The models were aligned pairwise by minimizing the normalized spatial discrepancy (NSD) score with the program SUPCOMB [35]. The mean NSD score of 0.75 (values ranging from 0.74 to 0.77) indicated an adequate convergence of the models [34]. All figures were generated with PyMOL (http://www.pymol.org).

### Crystallization, data collection and structure determination and refinement

Crystallization conditions were screened by the hanging drop vapor diffusion method using a PixSys4200 Cartesian robot (high-throughput crystallization laboratory at EMBL Grenoble, France). The screen was performed by combining 0.1 µl of protein solution at 8 mg.mL^−1^ in buffer A with 0.1 µl of Hampton Crystal Screen solutions. Hits were reproduced manually. The N_Δ21_^0^-P_60_ complex crystallized at 20°C in 0.1 M sodium acetate buffer, pH 4.6, containing 4% (w/v) of PEG4000. Single crystals were harvested from the drop, briefly soaked in the reservoir solution supplemented with 25% glycerol and flash frozen in liquid nitrogen at 100 K before data collection. X-ray diffraction data were collected at a wavelength of 0.933 Å on the ID14-2 beamline at the ESRF (Grenoble, France).

The data were processed using the program iMosflm [36] and scaled with the program Scala from the ccp4 suite [37]. The structure was solved by molecular replacement with the program Phaser [38] using residues 22 to 422 of a protomer of N (2GIC, chain E) extracted from the N-RNA crystal structure [15] as a search model. The visible part of P_60_ was assigned and constructed with the program Buccaneer [39], and the overall structure was refined to 3.0 Å resolution using Coot [40] and Refmac5 [41]. The quality of the model was checked with PROCHECK [42]. Data collection and refinement statistics are summarized in Table 1.

The N_Δ21_^0^-P_60_ complex was crystallized in space group P2_1_2_1_2. The crystallographic asymmetric unit contained five protomers of N_Δ21_^0^-P_60_ complex. Each protomer includes residues 22 to 422 of N and residues 6 to 33 of P_60_. In some protomers, residues 34 and 35 could also be constructed.

### NMR spectroscopy

NMR experiments were performed on a Varian spectrometer operating at a ^1^H frequency of 600 MHz. All samples contained 20 mM Tris-HCl, 150 mM NaCl, 50 mM Glu, 50 mM Arg with 10% D_2_O adjusted to pH 6.0. The concentration of free ^15^N, ^13^C, ^2^H-labeled P_60_ was 0.9 mM and the concentration of the N_Δ21_^0^-P_60_ complex (^15^N, ^13^C, ^2^H-labeled P_1–60_ in complex with unlabeled N) was 0.28 mM. In the titration experiment, ^15^N-labeled P_60_ was initially at 0.28 mM. MBP- N_Δ21_ was added at final concentrations of 0.09 mM or 0.17 mM. ^15^N *R*_2_ (CPMG) relaxation experiments were acquired using standard pulse sequences [43]. The spectra were acquired with a sweep width of 8.0 kHz and 512 complex points in the ^1^H dimension, and a sweep width of 1.2 kHz and 200 complex points in the ^15^N dimension. The magnetization decay was sampled at 10, 30, 50, 70, 90, 130, 170, 210 and 250 ms and the peak heights were used to extract the relaxation rates. To obtain estimates of the errors on the relaxation rates, a repeat measurement of one of the relaxation delays (70 ms) was carried out.

## Supporting Information

Text S1 This file contains seven additional figures named S1 to S7 and an additional table S1.
(DOC)
