# Supplementary material for: Structure of the Vesicular Stomatitis Virus N0-P Complex
Source: PLoS Pathog. 2011 Sep 22;7(9):e1002248. doi: 10.1371/journal.ppat.1002248 (PMC3178552; doi:10.1371/journal.ppat.1002248)
Supplement: Text S1 — This file contains seven additional figures named S1 to S7 and an additional table S1. (DOC) [file ppat.1002248.s001.doc]

SUPPORTING INFORMATION

**Structure of the VESICULAR STOMATITIS VIRUS N^0^-P complex**

Cédric Leyrat, Filip Yabukarski, Nicolas Tarbouriech, Euripedes de Almeida Ribeiro Jr, Malene Ringkjøbing Jensen, Martin Blackledge, Rob W. H. Ruigrok and Marc Jamin

**Supplementary figures**

**Figure S1.** Production and purification of MBP-N_Δ21_-RNA complexes. (A) Expression of MBP-N_Δ21_ in *E. coli* led to the formation of soluble particles that eluted mainly in a peak at 9.4 mL close to the exclusion volume (8.6 mL) of a Superdex S200 column. The collected fractions analyzed by denaturing gel electrophoresis are numbered at the bottom of the figure. (B) Denaturing 4-20 % gradient PAGE stained by Coomassie blue. Fraction numbers correspond to those indicated in fig. S1A. The gel shows the presence of a single protein below the 100 kDa marker in agreement with the molecular mass expected for the chimeric MBP-N_Δ21_ (88,326 Da). (C) Absorbance spectrum of the fraction eluting in the main peak (9.4 mL) indicating the co-elution of nucleic acid with the protein.

**±**

**±**

**±**

**Fig. S2.** Purification of the MBP-N_Δ21_^0^-P_60_ complex. (A) Overnight incubation of the MBP-N_Δ21_ complex with an excess of P_60_ led to the production of a new complex that eluted at 14.2 mL from the Superdex S200. The component eluting at 17.8 mL had a molecular mass of 9 ± 1 kDa and corresponded to the excess of P_60_. The collected fractions analyzed by denaturing gel electrophoresis are numbered at the bottom of the figure. (B) Denaturing 4-20% gradient PAGE stained with Coomassie blue. MBP-N_Δ21_ and P_60_ co-eluted in the peak at 14.2 mL (red boxes), while the peak at 17.2 mL contains only P_60_. Fraction numbers correspond to those indicated in Fig. S2A. (C) Absorbance spectrum of the fraction eluting in the peak at 14.2 mL indicating a lower nucleic acid content.

**A**

**B**

**C**

**D**

**E**

**Fig. S3.** Small-angle X-ray scattering experiments. (A) Scattering curve of N_Δ21_^0^-P_60_ recorded at different concentrations. Protein concentrations: 2.7 mg.mL^-1^ (black curve), 5.3 mg.mL^-1^ (red curve) or 8.0 mg.mL^-1^ (blue curve). (B) Guinier plot. (C) Distance distribution function. D_max_ values are shown in Table S1. The surface areas under the curves were normalized to account for the differences in protein concentration. (D) Kratky plot. The bell-shaped curves indicated a well-folded globular protein. (E) Modeling of SAXS data with DAMMIN. The theoretical SAXS curve derived from the bead model shown in Figure 2D (green line) reproduces the experimental SAXS data (open circles) up to 3.5 nm^-1^ (shown in Figure 2C).

**Fig. S4.** *Ab initio* bead models of the N_Δ21_^0^-P_60_ complex. (A) Comparison of the theoretical SAXS curve calculated for a N protomer extracted from the circular N-RNA complex (2GIC chain E) (red line) or for a N_Δ21_^0^-P_60_ protomer extracted from the circular complex (green line) with the experimental SAXS curve recorded for the N_Δ21_^0^-P_60_ complex (dots). The SAXS curve was recorded at ESRF beamline ID 14-3. (B) Plot of the residuals between the theoretical curve calculated for a N protomer extracted from the circular N-RNA complex and the experimental curve of the soluble complex. (C) Plot of the residuals between the theoretical curve calculated for a N_Δ21_^0^-P_60_ and the experimental curve. (D) Superposition of the average *ab initio* bead model of the N_Δ21_^0^-P_60_ complex with a N_Δ21_^0^-P_60_ protomer extracted from the atomic structure of the circular complex.

**Fig. S5.** Comparison of the structure of N in the N_Δ21_-P_60_ complex and in the N-RNA complex. The structure of N_Δ21_ and of P_60_ (aa 6-35) extracted from the decameric complex (chains E and N) are shown in green and red, respectively. The structures of N and of the bound RNA extracted from the decameric complex (2GIC chain E) are shown in yellow and blue, respectively. The r.ms.d. between the N molecule is 0.96 Å.

Figure S6. Sequence conservation between VSV (2GIC) and RAV N (2GTT). The alignment was performed with ClustalW2 and the figure was prepared with ESPript . The identities are displayed as white characters in a red box, while the similarities (using the Risler score ) are displayed in red. The blue box highlights the central hinge region (aa 200-300). The close arrows in dark and light blue show the identical and similar residues lining the binding groove of the MoRE of P (circled in black in figure 5B), respectively. The open arrows in dark and light blue show the identical and similar residues forming a hydrophobic surface of N that interacts with both P and the RNA. The arrows in magenta show the three residues of VSV N forming salt bridges with the MoRE of P (Figure 5A). The green arrows show the basic residues of VSV N that binds directly phosphate groups of the RNA. The close green arrows show the residues that are conserved in RAV.

**A**

**Fig. S7.** Superposition of the 5 protomers of P_60_ and of N_Δ21_ forming the asymmetric unit. (A, B) The overlay of the peptide moiety from the five protomers of the asymmetric unit revealed that residues 14 to 31 exhibit the lowest level of flexibility. The backbones of the different protomers take similar conformations but the side chains of residues 6 to 11 and of residues 32 to 35 take various orientations in the different protomers. The remaining residues (aa 1-5 and aa 36-68) are not visible in the crystal structure probably owing to their structural flexibility. (C) The different protomers of N_Δ21_ are very similar. The backbone structures are color coded according to the crystallographic B-factors ranging from blue (low B-factor values) to red (high B-factor value).

**B**

**B**

**Table S1.** Molecular dimensions of the N_Δ21_^0^-P_60_ complex calculated from SAXS data

| **Protein concentration (mg.mL^-1^)** | **R_g_  (Guinier) (nm)** | **D_max_   (nm)** |
| --- | --- | --- |
|  |  |  |
| 2.7 | 2.7 ± 0.1 | 9.3 |
| 5.3 | 2.7 ± 0.1 | 9.2 |
| 8.0 | 2.8 ± 0.1 | 9.4 |
|  |  |  |

**References**

1. Chenna R, Sugawara H, Koike T, Lopez R, Gibson TJ, et al. (2003) Multiple sequence alignment with the Clustal series of programs. Nucleic Acids Res 31: 3497-3500.

2. Gouet P, Robert X, Courcelle E (2003) ESPript/ENDscript: Extracting and rendering sequence and 3D information from atomic structures of proteins. Nucleic Acids Res 31: 3320-3323.

3. Risler JL, Delorme MO, Delacroix H, Henaut A (1988) Amino acid substitutions in structurally related proteins. A pattern recognition approach. Determination of a new and efficient scoring matrix. J Mol Biol 204: 1019-1029.
